# Early Detection and Treatment of Congenital Cataracts Using Fetal Ultrasound: A Case of a Newborn With a Family History of Congenital Cataracts

**DOI:** 10.7759/cureus.53189

**Published:** 2024-01-29

**Authors:** Megumi Ito, Takashi Negishi, Sachi Funayama, Satoko Murakami, Sachiko Iizuka

**Affiliations:** 1 Department of Ophthalmology, Juntendo University School of Medicine, Tokyo, JPN; 2 Department of Obstetrics and Gynecology, Nerima General Hospital, Tokyo, JPN; 3 Department of Ophthalmology, Nerima General Hospital, Tokyo, JPN

**Keywords:** genetic disorder, japan, prenatal diagnosis, ultrasonography, cataracts

## Abstract

This case study highlights the advances in fetal ultrasonography, illustrating its role in early detection and management of congenital cataracts. We present the case of a male infant with a family history of congenital cataracts, where an in-utero ultrasound examination at 25 weeks of gestation revealed potential cataracts. His mother and brother underwent cataract surgery. After birth examination revealed that the infant was diagnosed with bilateral congenital cataracts at two days. Bilateral lens aspiration and anterior vitrectomy without intraocular lens insertion were done. Postnatal examinations and surgical interventions, including bilateral lens phacoemulsification and anterior vitrectomy without intraocular lens insertion, were conducted. This study discusses the importance of early detection, especially in familial cases, and the role of prenatal and postnatal care in managing congenital cataracts. It underscores the need for collaboration between ophthalmologists and obstetricians and the value of psychological support for the parents. The findings advocate for proactive fetal monitoring, particularly in genetically predisposed cases, to facilitate early diagnosis and treatment planning.

## Introduction

Recent advances in fetal ultrasonography technology have made it possible to clearly visualize the fetal head and face [1-3]. Congenital cataracts are a rare condition where cloudiness forms in the lens of the eye at birth or shortly after [4]. They can occur in one or both eyes and can range from small areas of cloudiness to large opacities that interfere with vision [4]. As for how common they are, the incidence of congenital cataracts is estimated to be around 3-4 per 10,000 live births, making them relatively uncommon [4]. However, this can vary based on different populations and regions [4]. Screening for congenital cataracts often begins with routine examinations shortly after birth. Pediatricians and eye care specialists check for signs such as an abnormal red reflex during an eye examination, which could indicate the presence of cataracts [5]. Surgery for visually significant bilateral congenital cataracts is recommended to be performed in four to eight weeks as long as 10 weeks of age considering amblyopia and glaucoma [6].

Here, we report a case in which congenital cataracts were detected and treated as a result of an in-utero ultrasound examination of a fetus with a family history of congenital cataracts in the mother.

## Case presentation

General information

**Figure 1:**
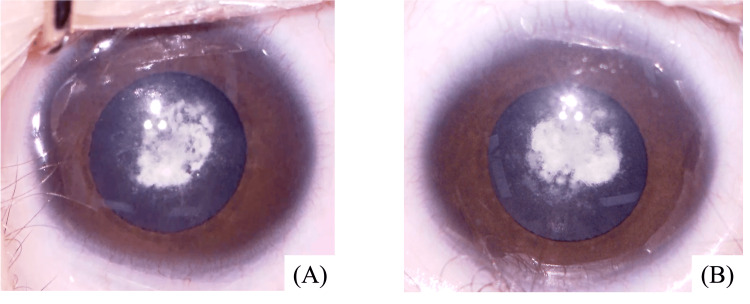
Preoperative microscope images (A) shows the right eye and (B) shows the left eye.

A 23-day-old boy presented with a congenital cataract. A fetal ultrasound at 25 weeks and three days of gestation indicated the possibility of congenital cataracts, and the baby was born by cesarean section at 38 weeks and zero days of gestation. He was diagnosed with congenital cataracts in both eyes by an ophthalmologist at two days of age in another hospital where he was born (Figure 1).

Family medical history

**Figure 2:**
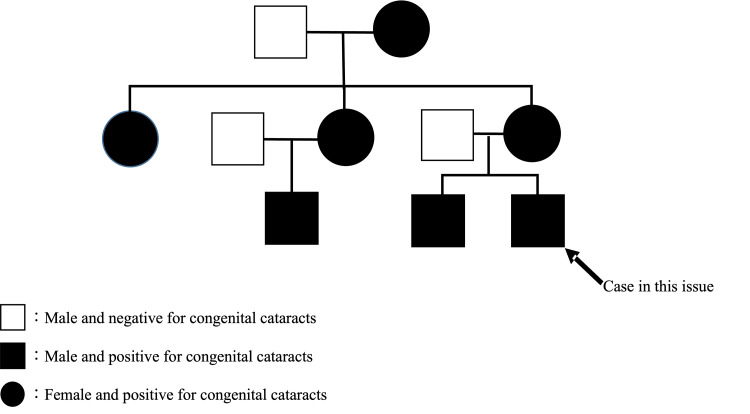
Pedigree chart. The grandmother of the present case is the proband, and congenital cataracts have developed in our patient’s mother’s siblings, indicating an autosomal dominant form of inheritance.

Figure 2 shows the family tree. Her older brother had congenital cataracts and underwent cataract surgery in both eyes at another hospital when he was five weeks old. The child’s mother, grandmother, aunt, and their children were also diagnosed with congenital cataracts. The mother had an aphakic eye complicated by glaucoma and had undergone surgery, while the grandmother and aunt were followed up without surgery. The father had multiple cystic kidneys. None of the close relatives had married each other.

Maternal medical history

The mother’s body mass index was 30.6 kg/m^2^ during maternity. Early pregnancy screening test showed rubella virus 32 times (HI method), and antibody tests for other infectious diseases were normal.

Fetal ultrasound findings

**Figure 3:**
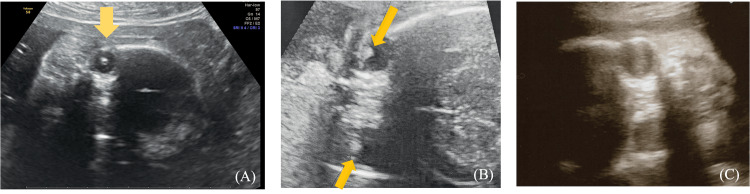
Fetal ultrasound image (25 weeks and three days in gestation). (A) Image from the frontal view of lens opacity highlighted with yellow arrows. (B) Image from the transverse view (scan) of lens opacity in both eyes highlighted with yellow arrows. (C) Fetal ultrasound image showing normal ultrasound findings at 26 weeks gestation as a reference image (25 weeks three days gestation). Normal lenses show white circles with a black center.

Fetal ultrasound at 25 weeks and three days of gestation showed echogenic findings in both eyeballs. (Figure 3).

Postnatal examination findings

**Figure 4:**
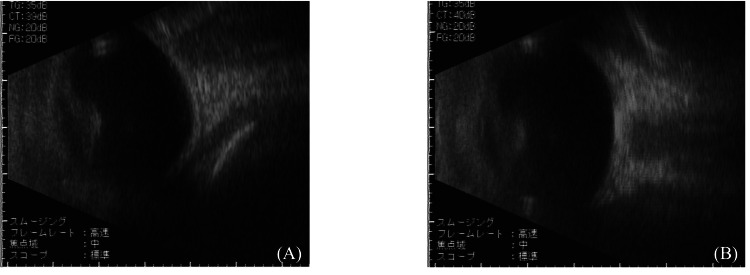
Ultrasonic B-mode photograph. (A) The right eye. (B) The left eye.

The birth weight was 3,760 g, and the auditory brainstem response and congenital metabolic screening tests were normal. The fundus of the eyes was difficult to see through, with strong nuclear cataracts in both lenses. Ocular ultrasound B-mode findings showed no obvious posterior abnormalities (Figure 4).

Progress

**Figure 5:**
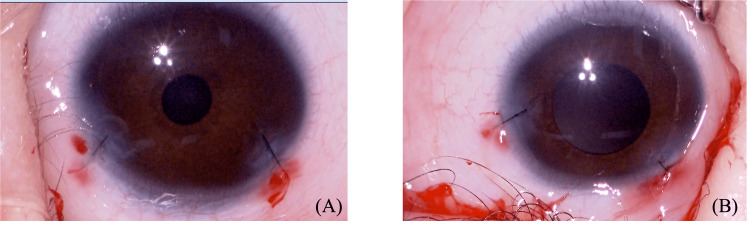
Postoperative image. (A and B) After aspiration of cataract and anterior vitrectomy.

At 12 weeks and six days of age, the patient underwent bilateral lens phacoemulsification and anterior vitrectomy under general anesthesia, and the surgery was completed without the insertion of an intraocular lens (Figure 5). Intraoperative fundus examination revealed no obvious fundus disease. Seven days after surgery, the refraction was measured, and glasses with +24.0 D on both eyes were prescribed. At 15 months after the surgery, an increase in intraocular pressure up to 40 mmHg was observed with the use of a tonometer (Icare® (Icare Finland Oy, Finland)) and ripasudil hydrochloride hydrate eye drops were started.

## Discussion

The prevalence of congenital cataracts is approximately 4.24 per 10,000 live births [7]. It has a wide variety of causes, including idiopathic, hereditary, infection in utero, drug-induced, and systemic diseases, with most hereditary cases being bilateral and most idiopathic cases unilateral [7].

Fetal lens development begins at three or four weeks of gestation and can be detected by ultrasonography as early as 13 weeks of gestation [8]. In normal lenses, the lens appears as an ellipsoid with high echogenicity at the limbus and uniformly low echogenicity in the interior, but in cataracts, there is a uniform opacity of the lens and loss of the central hypoechoic area [9]. Fetal ultrasonography has recently been able to detect not only cataracts but also lacrimal hernia, microphthalmia, anophthalmia, retinoblastoma, interocular narrowing, interocular divergence, etc., owing to advances in diagnostic imaging [8].

Table 1 summarizes previously reported cases of congenital cataracts diagnosed by fetal ultrasonography since 2000 [7,10-17]. Most of the cases were bilateral with a family history, and the diagnosis was made in the second trimester of pregnancy. Postnatal examination revealed cataracts consistent with prenatal findings in all cases, suggesting that fetal ultrasonography is useful. However, evaluation early after birth is necessary because the presence of small corneal or ocular fundus complications cannot be determined in the fetal period.

**Table 1:** Previously reported cases of congenital cataracts diagnosed by fetal ultrasonography since 2000.

Author	Number of cases	Cataracts	Family history of cataracts	Weeks at diagnosis (week)	Ocular disease	Systemic diseases
Jung et al. [7]	8	Binocular 7/8	Yes 3/8	Average 26	Yes 3/8	Yes 1/8
Mashiach et al. [10]	4	Binocular 3/4	Yes 4/4	Average 15	-	-
Reches et al. [11]	1	Binocular 1/1	Yes 1/1	Average 24	-	Yes 1/1
Lee et al. [12]	1	Binocular 1/1	Yes 1/1	Average 36	-	-
Cengiz et al. [13]	1	Binocular 1/1	No	Average 20	-	-
Daskalakis et al. [14]	1	Binocular 1/1	No	Average 22	-	-
Chen et al. [15]	1	Binocular 1/1	No	Average 22	-	-
Aksay et al. [16]	1	Binocular 1/1	No	Average 21	-	-
Zheng et al. [17]	1	Binocular 1/1	No	Average 24	-	-
Qin et al. [18]	41	Binocular 32/41	Yes 6/41	Average 27	Yes 10/41	Yes 24/41

Several challenges may exist in the fetal diagnosis of congenital cataracts. The first is that the detection rate depends on the performance of the examination equipment and the skill of the examiner [18,19]. The second is that the main purpose of antenatal checkups in Japan is to confirm the health and development of the mother and fetus, not to detect fetal morphological malformations [20].

Early detection and surgery are necessary to treat congenital cataracts. Although it is difficult to screen all fetuses, it is important to suspect congenital cataracts on ultrasound when there is a family history of the condition to plan for treatment after delivery. For this reason, cooperation between obstetrics and ophthalmology is necessary, and ophthalmologists should request obstetricians to perform orbital delineation during mid-pregnancy examination.

## Conclusions

In our case, confirmed heredity led to a careful ultrasound examination at 25 weeks which revealed characteristic findings. Ophthalmoscopically, the diagnosis was confirmed at two days of age, and lens extraction could be performed at 12 weeks of age. These results suggest that the patient was diagnosed early, and planned treatment was possible because of the ophthalmology consultation soon after birth based on the in-utero diagnosis.
